# Layered Fusion Reconstruction Based on Fuzzy Features for Multi-Conductivity Electrical Impedance Tomography

**DOI:** 10.3390/s24113380

**Published:** 2024-05-24

**Authors:** Zeying Wang, Jiaqing Li, Yixuan Sun

**Affiliations:** 1 School of Mechanical Engineering, University of Shanghai for Science and Technology, Shanghai 200093, China; 2 School of Energy and Power Engineering, University of Shanghai for Science and Technology, Shanghai 200093, China; 2135051320@st.usst.edu.cn

**Keywords:** layered fusion, electrical impedance tomography (EIT), multi-conductivity distribution, local anomaly detection

## Abstract

In medical imaging, detecting tissue anomalies is vital for accurate diagnosis and effective treatment. Electrical impedance tomography (EIT) is a non-invasive technique that monitors the changes in electrical conductivity within tissues in real time. However, the current challenge lies in simply and accurately reconstructing multi-conductivity distributions. This paper introduces a layered fusion framework for EIT to enhance imaging in multi-conductivity scenarios. The method begins with pre-imaging and extracts the main object from the fuzzy image to form one layer. Then, the voltage difference in the other layer, where the local anomaly is located, is estimated. Finally, the corresponding conductivity distribution is established, and multiple layers are fused to reconstruct the multi-conductivity distribution. The simulation and experimental results demonstrate that compared to traditional methods, the proposed method significantly improves multi-conductivity separation, precise anomaly localization, and robustness without adding uncertain parameters. Notably, the proposed method has demonstrated exceptional accuracy in local anomaly detection, with positional errors as low as 1% and size errors as low as 33%, which significantly outperforms the traditional method with respective minimum errors of 9% and 228%. This method ensures a balance between the simplicity and accuracy of the algorithm. At the same time, it breaks the constraints of traditional linear methods, struggling to identify multi-conductivity distributions, thereby providing new perspectives for clinical EIT.

## 1. Introduction

Electrical impedance tomography (EIT) is a functional imaging technique that measurements boundary voltages to continuously reconstruct and visualize the conductivity distribution within an observed object [1,2]. This technique has several advantages, including safety, absence of radiation, rapid response, portability, and cost-effectiveness, rendering it a compelling choice for a variety of applications [3]. Consequently, EIT has attracted attention and has been widely employed across numerous fields. In particular, EIT is recognized for its utility in industrial measurements and biomedical detection. It is especially suitable for applications such as multi-phase flow visualization [4,5], in addition to lung perfusion monitoring [6,7], tactile perception [8], and gesture recognition [9,10].

The primary focus of EIT research in the medical field has been on the application of lung tomography as a biosensor [11,12]. This is due to the valuable diagnostic information that can be observed when biological tissues and organs exhibit significant changes in conductivity, corresponding with changes in physiological conditions such as respiration and blood flow [13]. A variety of lung lesions, including lung collapse, pneumothorax, pulmonary edema, and lung tumors, serve as clear indicators of abnormal air or fluid content within the lungs [14]. For instance, the pleural effusion model in EIT is characterized by regions of high conductivity (1.5 S/m at 100 kHz, approximately 7 times higher than the normal lung conductivity), resulting from the accumulation of fluid in the pleural space [13,15,16]. Similarly, tumor tissue has been observed to possess a significantly higher conductivity compared to normal lung tissue [17].

Mathematically, image reconstruction in EIT involves solving a highly nonlinear and ill-posed inverse problem from noisy voltage data [18]. This inherent limitation often results in low resolution and instability of reconstructed images, as well as high sensitivity to minor perturbations induced by noise and modeling errors. Consequently, while the capacity of EIT to reconstruct two-phase distributions, such as monitoring lung respiration, is deemed acceptable, it struggles when multiple field change media are present, such as when local lesions occur [17,19]. In such scenarios, minor local changes within a large target tend to be mixed by this large target, making it challenging to capture local anomalies within the lungs.

Nonlinear methods are directly applicable to nonlinear EIT problems. In recent years, deep learning has been widely investigated in the EIT domain owing to its proficiency in solving nonlinear problems, which broadly falls into three categories [20,21,22]. The first uses an approximation from voltage of conductivity for image mapping [23,24]. The second pre-reconstructs low-resolution images, then uses neural networks for high-res post-processing [25]. The third formulates the problem in an optimization framework, e.g., with neural networks as regularizers [15]. These methods provide precise and resilient reconstructions of lungs with local anomalies, as validated in both simulated and experimental environments. Given ample data, it is possible for learning-based methods to effectively reconstruct complex lung images in the clinic. However, these methods often face high time costs during the both training phase and the inference phase, lack physical interpretability, and struggle with objects significantly different from the training data.

Linearization methods for EIT are time-efficient, stable, and simple, making them prevalent in clinical practice [26,27,28,29]. Recent studies have demonstrated that the reconstruction accuracy of two-phase distributions, based on regularization algorithms, can be significantly enhanced through simple post-processing or a few iterative steps [30,31]. However, for the distribution of three or more phases, specifically in detecting local lung anomalies, it is still difficult for these methods to reconstruct conductivity distributions efficiently. It has been verified that incorporating prior information can enhance the detection of local lung anomalies [17,32]. Effective reconstruction of multi-conductivity distributions can also be accomplished by employing multi-modal fusion techniques, e.g., the fusion of frequency-difference and time-difference reconstructions [33]. However, these methods rely on either sufficient prior information or additional measuring procedures. Although EIT is designed to be fast, robust, versatile, and precise, current nonlinearization methods struggle with interpretability, time costs, and robustness, whereas linearization methods are unable to satisfy both the necessary simplicity and accuracy requirements simultaneously.

This work aims to improve the imaging capability of the linearization method in multi-conductivity EIT, maintaining its speed and simplicity. The EIT images reconstructed by the linearization method have the features of a fuzzy boundary, similar boundaries for different objects, and local unrecognizability. Based on these fuzzy features and the internal relationship of the EIT reconstruction process, a layered fusion reconstruction method is proposed, which adopts the strategy of difference decomposition and fusion. The proposed method strikes a balance between the simplicity and accuracy of EIT reconstruction, while simultaneously digging deeper into the potential of linearization methods through the inherent relationships and features of EIT.

The remainder of this paper is structured as follows. Section 2 describes the mathematical model of EIT, detailing its principles and equations. Section 3 introduces the proposed method of layered fusion based on EIT features. This section describes the fuzzy features, definition of EIT layers, reconstruction anomalies, and the layered fusion algorithm. Section 4 analyzes the performance and implications of the simulation and experimental results. Section 5 discusses the results. Finally, Section 6 draws conclusions from the study.

## 2. Mathematical Model of EIT

**Figure 1:**
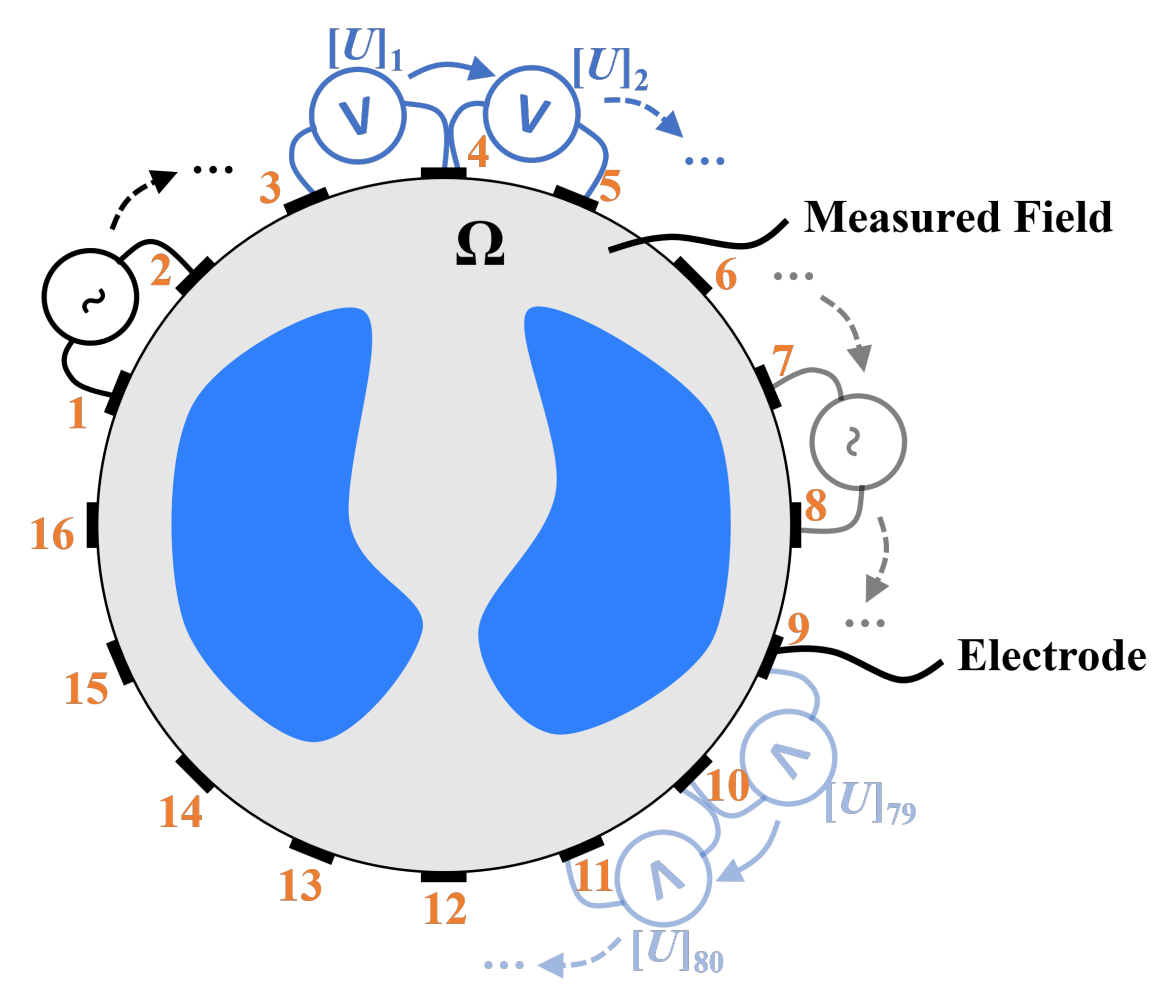
Principle of detection in EIT.

An EIT measurement system is designed to reconstruct the conductivity distribution within a domain Ω. The electrodes, as shown in Figure 1, are evenly distributed around an observed object. The data acquisition strategy of adjacent current excitation and adjacent voltage measurements is typically adopted.

The process begins with the application of current excitation to one adjacent electrode pair as the initial excitation electrode, with 1 and 2 being selected as the first pair of the excitation current source in this example. Subsequently, two adjacent electrodes are selected successively to measure voltages as the measurement electrode, such as 3–4, 4–5, ..., 14–15, 15–16. This process successively uses 16 pairs of adjacent electrodes as excitation electrodes.

Upon completion of a full measurement cycle, a boundary voltage vector is derived from $n_{E}$ electrodes, comprising $N=n_{E}\left(n_{E}-3\right)$, with the understanding that only half of these elements are independent. For instance, a total of 208 measurements can ultimately be collected from 16 electrodes. Collectively, these measurements can be used to describe one frame of a cross-section, providing a detailed description of the electrical properties of the subject. For the human body, these measurements reflect the underlying conductivity distribution, indicating physiological properties.

Given a known conductivity distribution and a specified excitation pattern, the forward problem is tasked with computing the electric potential and the boundary voltages. The observation model incorporates an additive Gaussian model to account for measurement errors. Partitioning the observation domain into *M* discrete pixels, the forward model is formulated as

(1) U=F(σ(Ω),I)+e

where F(·) represents an operator mapping the inner conductivity distribution $\sigma \left(\Omega \right)\in R^{M\times 1}$ and excitation current $I\in R^{N\times 1}$ into a boundary voltage vector $U\in R^{N\times 1}$ and $e\in R^{N\times 1}$ represents the additive noise. This question is typically solved using the finite element method (FEM) or the boundary element method (BEM).

With the reference conductivity $\sigma_{0}$, Equation (1) can be expanded using the first-order Taylor series as

(2) $$U=U_{0}+S\left(\sigma_{0}\right)\left(\sigma -\sigma_{0}\right)+O\left(\sigma \right)$$

where $S\left(\sigma_{0}\right)=\frac{\partial {\left[U_{0}\right]}_{i}}{\partial {\left[\sigma_{0}\right]}_{j}}{|}_{\sigma =\sigma_{0}}$ is the Jacobin matrix, representing the differential of the boundary voltage to the conductivity.

After discrete approximation, the mathematical model of the EIT inverse problem can be linearly expressed as

(3) ΔU≈SΔσ+e

where $\Delta U=U-U_{0}$, representing the difference between the boundary voltage data in measured state and reference state, and $\Delta \sigma =\sigma -\sigma_{0}$, representing the difference between the measured state and reference state.

This process is inherently ill posed and ill conditioned, which often results in unreliable and unstable solutions. The inverse problem is typically solved using the least squares error method. However, to prevent overfitting in the least squares problem, regularization-based algorithms are commonly employed. The objective function of the regularization can be formulated as

(4) $$\Delta \sigma ={\arg\min}_{\Delta \sigma \in R^{M\times 1}}\{||S\Delta \sigma -{\Delta U||}_{2}^{2}+R\left(\Delta \sigma \right)\}$$

where the regularization term R(σ) commonly uses the $L_{2}$-norm as

(5) $$\Delta \sigma ={\arg\min}_{\Delta \sigma \in R^{M\times 1}}\{||S\Delta \sigma -{\Delta U||}_{2}^{2}+{\lambda ||\Delta \sigma ||}_{2}^{2}\}$$

where λ denotes the regularization parameter. Tikhonov-type regularization is a commonly used method and employs an analytic expression to obtained the estimation as

(6) $$\Delta \tilde{\sigma }={\left(S^{T}S+\lambda L^{T}L\right)}^{-1}S^{T}\Delta U$$

where **L** represents the regularization matrix, which is typically configured as a unit matrix.

Regularization methods are usually fast and relatively accurate. However, in the reconstruction process of multiple conductivity domains, there is a complex intermingling of both normal and abnormal objects. These objects lack clear demarcations under most typically used reconstruction methods. For practical applications, it becomes imperative to segregate these local anomalies. This segregation allows for a more accurate interpretation and analysis.

## 3. Layered Fusion Based on EIT Features

### 3.1. Fuzzy Features of Multi-Conductivity Distributions

With traditional reconstruction, as well as further operations such as iteration or filtering, it is possible to accurately reconstruct domains with binary conductivity using EIT. However, linearization methods generally lack the ability to reconstruct multi-conductivity distributions.

**Figure 2:**
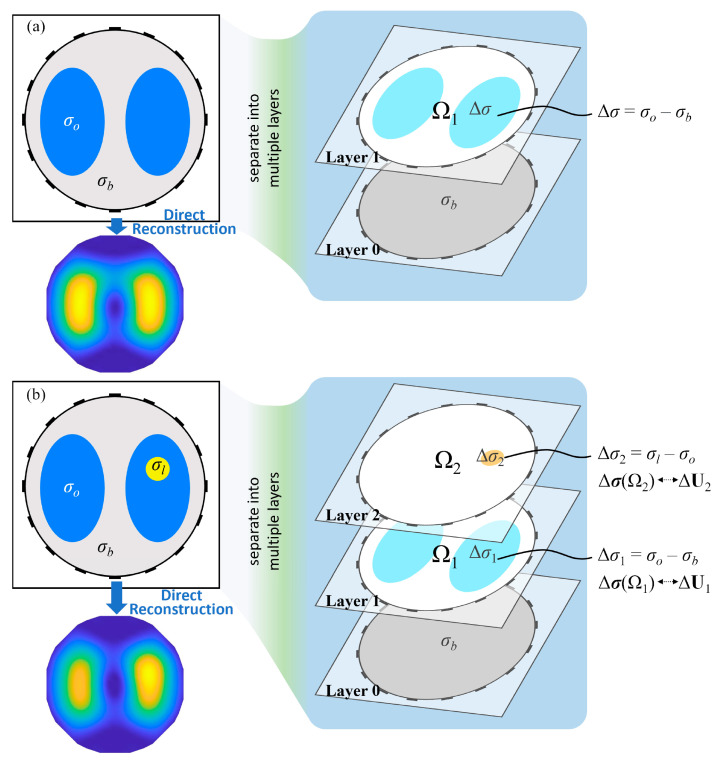
Layers in observed domains with binary and multiple conductivity distributions. (**a**) Binary conductivity distribution decomposed into a two-layer domain. (**b**) Multi-conductivity distribution decomposed into a three-layer domain.

In Figure 2a, there is an EIT observed cross-section with two main objects, and the right object in Figure 2b features a local anomaly area. The background, the objects and the anomaly area (e.g., a lesion in the lung) have different conductivities. It is assumed that there is no prior information on the internal distribution. The goal is to reconstruct the abstract distribution $\Delta \sigma =\sigma -\sigma_{ref}$, where $\sigma_{ref}$ is the uniform conductivity distribution with background conductivity $\sigma_{b}$.

Typical $L_{2}$-norm regularization in Equation (6) is adopted to directly reconstruct the conductivity distribution. Intuitively, from the reconstructed result $\Delta \tilde{\sigma }$ in Figure 2b, the main observed objects are not recognizable. Specifically, when comparing the two imaging results, it can be observed that the direct imaging results of the multi-conductivity distribution have the following features:

- A fuzzy boundary: In multi-conductivity distribution results, the boundaries exhibit significant ambiguity. This is observed both at object–background and lesion–object interfaces. The indistinct boundaries complicate element differentiation and add complexity to image interpretation.
- Similar boundaries for different objects: Consider two main objects in the multi-conductivity result. One is uniformly distributed, while the other exhibits local anomalies. Despite these differences, both objects share a common trait: their boundaries are similar. In other words, the presence of local anomalies does not significantly alter the main boundary.
- Local unrecognizability: The local conductivity variations within the object are merged into changes in the overall object conductivity. Despite processing, it is difficult to recognize the local changes in size and position.

Any multi-conductivity distribution can be regarded as a series of multiple binary conductivity distribution layers. In Figure 2a, the observed domain with binary conductivity can be divided into two layers. These correspond to the uniformly distributed reference and the imaging goal, which is represented as a change in conductivity. In Figure 2b, the observed domain with three conductivity values can be divided into three layers. Each of these layers contains only one main conductivity value. Layer 0 corresponds to the reference $\sigma_{ref}$. According to Equation (3) and ignoring the noise effect, the linearized model of Layer 1 can be expressed as

(7) $$\Delta U_{1}\approx S\Delta \sigma \left(\Omega_{1}\right)$$

where $\Delta \sigma (\Omega_{1})$ is the conductivity distribution of Layer 1, and its normalized reconstructed approximation is $\Delta {\tilde{\sigma }}_{1}$.

The linearized model of Layer 2 is

(8) $$\Delta U_{2}\approx S\Delta \sigma \left(\Omega_{2}\right)$$

where $\Delta \sigma (\Omega_{2})$ is the conductivity distribution of Layer 2, and its normalized reconstructed approximation is $\Delta {\tilde{\sigma }}_{2}$.

The combination of Layer 1 and Layer 2 corresponds to the abstract distribution as

(9) $$\Delta \sigma =\Delta \sigma \left(\Omega_{1}\right)+\Delta \sigma \left(\Omega_{2}\right)$$

Once the conductivity distribution for each layer has been obtained, an approximation of the total conductivity distribution can then be calculated by summing up the layers. To determine the distribution of the layers, voltage changes need to be calculated first.

According to the feature analysis of a multi-conductivity distribution, the boundaries of the main objects are similar in the reconstruction results, regardless of the presence of anomalies. Therefore, based on the object recognition threshold [26], the main objects can be filtered from the reconstructed result. Consequently, Layer 1 can then be obtained as

(10) $${\left[\Delta {\tilde{\sigma }}_{1}\right]}_{i}=\left\{\begin{array}{cc} 1, & \mathrm{if}{\left[\Delta \tilde{\sigma }\right]}_{i}\geq \frac{1}{4}\max\left({\left[\Delta \tilde{\sigma }\right]}_{i}\right)\\ 0, & \mathrm{otherwise}. \end{array}\right.$$

where $\Delta {\tilde{\sigma }}_{1}$ contains all pixels ${\left[\Delta \tilde{\sigma }\right]}_{i}$ greater than $\frac{1}{4}$ of the maximum amplitude.

$\Delta {\tilde{\sigma }}_{1}$ is the normalized result, and the real conductivity is unknown without prior information, so the actual distribution implies a known scale factor set to *a*. According to Equation (7), the voltage change of Layer 1 $\Delta {\tilde{U}}_{1}$ can be approximated by

(11) $$\Delta {\tilde{U}}_{1}\approx S\left(a\Delta {\tilde{\sigma }}_{1}\right)=a\left(S\Delta {\tilde{\sigma }}_{1}\right)$$

In the linearized EIT model, the voltage change of Layer 2 $\Delta {\tilde{U}}_{2}$ can be obtained by the difference between the total change and the change of Layer 1 as

(12) $$\Delta {\tilde{U}}_{2}\approx \Delta U-\Delta {\tilde{U}}_{1}$$

The current problem is that the scale factor *a* related to the voltage change set is still unknown.

### 3.2. Features of Measurement Changes

**Figure 3:**
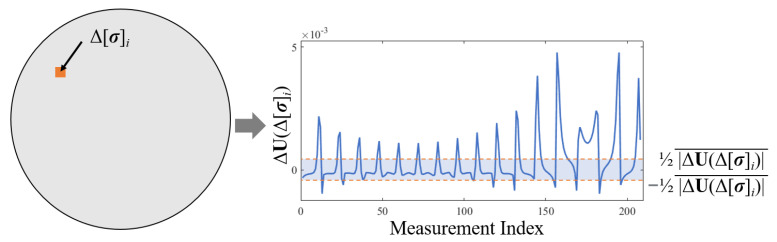
Sensitivity analysis of measurement variations influenced by local conductivity changes, with the low-sensitivity range indicated.

According to the definition of sensitivity, the sensitivity of measurements is influenced by changes in conductivity. Figure 3 illustrates the variations in measurements resulting from a unit change in conductivity. In the linearized EIT model, the sensitive responses from larger areas of local conductivity changes are additive. Among these variations, there always exists a portion with very low changes in measurements, termed as low-sensitivity ranges.

The threshold is set to $\pm \frac{1}{2}\bar{\left|\Delta U\right|}$, where $\bar{\left|\Delta U\right|}$ is the average of the absolute values of the measurement set. When conductivity distribution changes are small and local abnormal, as in Figure 2, the voltage measurements in these low-sensitivity ranges remain almost constant. This implies that the scale of the low-sensitivity range is nearly invariant. To quantify this scale, a low-sensitivity range set is defined as

(13) $$U_{L}=\{{\left[\Delta U_{b}\right]}_{i}\left|\right|{\left[\Delta U_{a}\right]}_{i}\left|<\frac{1}{2}\bar{|\Delta U_{a}|}\right\}$$

where $U_{L}$ represents the set of observed voltage changes ${\left[\Delta U_{b}\right]}_{i}$ that correspond to the low-sensitivity range, where the absolute values of the initial measurement changes ${\left[\Delta U_{a}\right]}_{i}$ are less than half the average absolute change $\bar{|\Delta U_{a}|}$.

Furthermore, to calculate the scale of the low-sensitivity range of $\Delta U_{b}$ at the initial measurement change $\Delta U_{a}$, the scale feature is expressed as an operator L(·,·) as

(14) $$L\left(\Delta U_{b},\Delta U_{a}\right)=\frac{1}{l}\sum_{j=1}^{l}\left|{\left[U_{L}\right]}_{j}\right|$$

where *l* represents the total number of elements within the low-sensitivity range and ${\left[U_{L}\right]}_{j}$ represents the *j*th element in the observed voltage change set $\Delta U_{b}$ that corresponds to measurements in $\Delta U_{a}$ falling within the low-sensitivity range.

Taking into account the size ratio of the tumor to the chest and referring to the conductivity of each tissue at a frequency of 100 kHz, the overall difference in conductivity between Layer 1 and the whole is about 0.1 S/m [13]. Compared to the overall conductivity of the chest (approximately 2 S/m), such a conductivity difference makes the corresponding voltage changes approximately the same. Setting the overall measurement set ΔU as the initial change, the proportional coefficient *a* can be expressed as

(15) $$a\cdot L(\frac{1}{a}\Delta {\tilde{U}}_{1},\Delta U)=L(\Delta U,\Delta U)$$

Combining Equations (11) and (15), $\Delta {\tilde{U}}_{1}$ can be computed by

(16) $$\Delta {\tilde{U}}_{1}\approx \frac{L(\Delta U,\Delta U)}{L(S\Delta {\tilde{\sigma }}_{1},\Delta U)}S\Delta {\tilde{\sigma }}_{1}$$

The voltage change of Layer 2 can be obtained from Equations (12) and (16). Then, the local anomalies on Layer 2 can be reconstructed by Equation (6). For the purpose of clearer boundaries, the reconstruction can be filtered by

(17) $$f\left({\left[\Delta {\tilde{\sigma }}_{2}\right]}_{i}\right)=\left\{\begin{array}{cc} {\left[\Delta {\tilde{\sigma }}_{2}\right]}_{i}, & \mathrm{if}\;{\left[\Delta {\tilde{\sigma }}_{2}\right]}_{i}\geq \frac{1}{4}\max\left(\Delta {\tilde{\sigma }}_{2}\right)\\ 0, & \mathrm{otherwise}. \end{array}\right.$$

If an approximate proportion of anomaly area *p* is known, the conductivity change in Layer 2 can be calculated more accurately. The first step is to sort $\Delta {\tilde{\sigma }}_{2}$ in order from largest to smallest as $\Delta {\tilde{\sigma }}_{2}^{\prime }$. Then, the the conductivity change can be rebuilt as

(18) $${\left[\Delta {\tilde{\sigma }}_{2}^{\prime \prime }\right]}_{i}=\left\{\begin{array}{cc} {\left[\Delta {\tilde{\sigma }}_{2}\right]}_{i}, & \mathrm{if}\;{\left[\Delta {\tilde{\sigma }}_{2}\right]}_{i}\geq {\left[\Delta {\tilde{\sigma }}_{2}^{\prime }\right]}_{t}\\ 0, & \mathrm{otherwise}. \end{array}\right.$$

where t=⌊p×M⌋ represents the number of elements in the anomaly area.

### 3.3. Layered Fusion Algorithm

The conductivity change for each layer has been reconstructed and normalized. If the real conductivity of each area is known, it can be directly superimposed in proportion through the relationship of conductivity values across layers. However, in practice, the real conductivity is difficult to ascertain accurately. One approach is to roughly assign proportions based on a prior range, while another is to estimate the conductivity range based on the scale of voltage changes.

When the conductivity change is known, the final conductivity change result can be directly calculated by adding the proportional layers as

(19) $$\Delta {\tilde{\sigma }}^{\prime }=\delta \sigma_{1}\Delta {\tilde{\sigma }}_{1}+\delta \sigma_{2}\Delta {\tilde{\sigma }}_{2}^{\prime \prime }$$

Prior information is usually lacking; thus, $\delta \sigma_{1}$ and $\delta \sigma_{2}$ are unknown. In this case, it is necessary to estimate the ratio between the conductivity change of the different layers. The conductivity distribution of Layer 1 and Layer 2 changes uniformly within observed objects. Such a conductivity change set can be expressed as

(20) $$\Delta \tilde{\sigma }=\alpha \cdot {\left[0,0,\ldots ,1,1,\cdots ,0,0,\ldots \right]}^{T}$$

where α represents the real conductivity change value. This means $\Delta \tilde{\sigma }$ can be regarded as a number times a binary matrix. Then, the indices of all working elements can be represented as

(21) $$I=\{i|{\left[\Delta \tilde{\sigma }\right]}_{i}=\alpha ,i\in \left\{1,2,\ldots ,M\right\}\}$$

It can be deduced that the measurement change is actually the superposition of the effective columns of the sensitivity matrix, which can be expressed according to Equation (3), ignoring the noise effect, as

(22) $$\Delta U=\alpha \cdot (s_{{\left[I\right]}_{1}}+s_{{\left[I\right]}_{2}}+\cdots +s_{{\left[I\right]}_{n}})$$

where *n* is the number of working elements and $s_{{\left[I\right]}_{1}},s_{{\left[I\right]}_{2}},\cdots ,s_{{\left[I\right]}_{n}}$ represent the working columns of the matrix **S**.

Since the sum of each column of the normalized sensitivity matrix is 1, the mean of the voltage change set can be calculated by

(23) $$mean\left(\Delta U\right)=\frac{1}{N}\sum_{i=1}^{N}{\left[\Delta U\right]}_{i}=\frac{1}{N}\cdot \alpha \cdot n$$

In the layered model, Equation (23) can be rewritten as

(24) $$mean\left(\Delta {\tilde{U}}_{1}\right)=\frac{1}{N}\cdot \delta \sigma_{1}\cdot n_{1}$$

(25) $$mean\left(\Delta {\tilde{U}}_{2}\right)=\frac{1}{N}\cdot \delta \sigma_{2}\cdot n_{2}$$

where $n_{1}$ and $n_{2}$ represent the number of pixels in working areas of Layer 1 and Layer 2, respectively. Specifically, $n_{1}$ is the total number of pixels at an estimated state of 1 on Layer 1, calculated by Equation (10), and $n_{2}$ can be estimated by the sum of the normalized distribution of Layer 2. In this way, the fusion weights $\delta \sigma_{1}$ and $\delta \sigma_{2}$ can be obtained, and Layer 1 and Layer 2 can then be fused by Equation (19).

**Figure 4:**
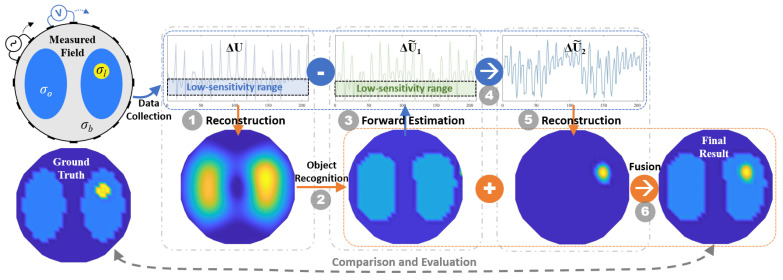
Visualized workflow of the layered fusion framework for multi-conductivity EIT.

Algorithm 1 describes the pseudo-code implementation of the proposed layered fusion (LF) reconstruction. Figure 4 illustrates the overall process of the layered fusion reconstruction approach, in which step numbers are marked in the corresponding processing. During the initialization period, the regularization parameter λ can be obtained in advance through the L-curve method, GCV, unsupervised evaluation optimization or other methods, and then calculated using unified parameters. The proportion of the abnormal area *p* is an optional input. The proposed method avoids any tedious parameter adjustment. The final result is obtained through layered processing and then fusion based on EIT internal features and fuzzy features. Details on the comparison and evaluation will be further discussed in the subsequent section.
**Algorithm 1** Algorithm flow of LF reconstruction.**Input**: Voltage measurement: ΔU; Sensitivity matrix: S; Regularization parameter: λ; Proportion of anomaly area *p*.**Output**: Conductivity Set: $\Delta \tilde{\sigma }$.**Begin**  **Step 1**: Reconstruct the initial $\Delta \tilde{\sigma }$ using Equation (6);  **Step 2**: Estimate the binary distribution of Layer 1 $\Delta {\tilde{\sigma }}_{1}$ by Equation (10);  **Step 3**: Estimate the voltage change in Layer 1 $\Delta {\tilde{U}}_{1}$ by Equations (13), (14) and (16);  **Step 4**: Calculate the voltage change in Layer 2 $\Delta {\tilde{U}}_{2}$ by Equation (12);  **Step 5**: Reconstruct the conductivity distribution of Layer 2 $\Delta {\tilde{\sigma }}_{2}$ by Equation (6), and sharpen $\Delta {\tilde{\sigma }}_{2}$ according to existing conditions:          **if** *p* is known **then**                Filter the distribution by Equation (18)          **else**                Filter the distribution by Equation (17)          **end if**  **Step 6**: Fusion multiple layers $\Delta {\tilde{\sigma }}_{1}$ and $\Delta {\tilde{\sigma }}_{2}^{\prime \prime }$ by Equations (19),  (24) and (25).**End**

## 4. Experiments and Results

To investigate the reconstruction performance of the proposed layered fusion (LF) method, a set of simulation and experiment related to lung monitoring with anomaly area were performed.

### 4.1. Simulation Configuration

**Figure 5:**
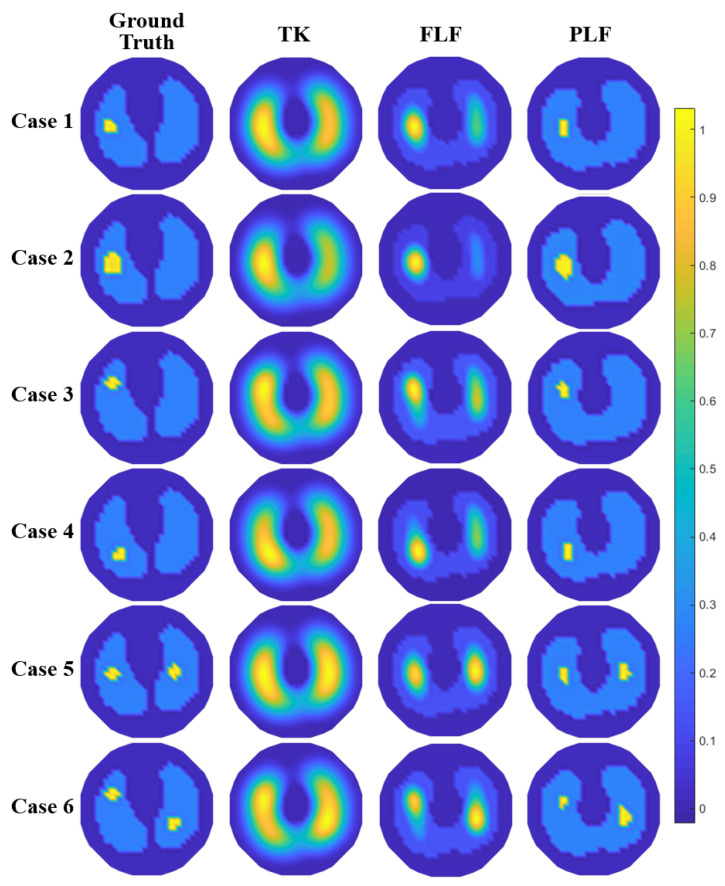
Comparative reconstruction results of simulation cases between methods of Tikhonov-type regularization (TK), filter-based layered fusion (FLF), and proportional-based layered fusion (PLF).

In simulations, a simplified thoracic sensor with N = 16 electrodes was built in COMSOL Multiphysics; the radius of the circular domain was 20 cm and the width of each electrode was 1 cm. An adjacent sensing strategy, as shown in Figure 1, was adopted. Each data frame consists of N(N−3)=208 measured values. Each EIT image contains 32 × 32 pixels. Six simplified lung-like phantoms in simulations are shown in the first column of Figure 5, which includes the tissues of lung and inner anomaly areas as lesions with various sizes or positions. The frequency and amplitude of the injected current were set as 100 kHz and 5 mA, respectively. Under this frequency, the conductivity of the lung was assigned as 0.27 S/m as a deflated state, and that of the anomaly was set as 0.6 S/m, based on findings that lung cancer tissue is 1.6 to 3.3 times more conductive than normal lung tissue [13,17].

The uniform distribution was set as a reference frame, and the abnormal lungs were directly adopted as imaging frames. To investigate the robustness of the proposed algorithm, signal-independent zero-mean additive white Gaussian noise was added to the voltage change set. To evaluate the effectiveness and robustness of the proposed algorithm, noise-free EIT data and data with a signal-to-noise (SNR) ratio of SNR = 30, 40, 50, 60, 70, 80 dB were collected. The SNR is expressed as

(26) $$\mathrm{SNR}=10\mathrm{lg}(\frac{P_{u}}{P_{noise}})$$

where $P_{u}$ represents the power of a frame of measurement data and $P_{noise}$ represents the power of the corresponding Gaussian noise.

For the purpose of assessing the effectiveness of the proposed method, typical Tikhonov regularization (TK) was adopted for a visual comparison. In order to simulate the actual situation, the proportion parameter *p* of each phantom was collected. Considering two rather extreme cases, without any prior information and with prior known *p* and conductivity ratio, direct filtering LF (FLF) based on Equation (17) and proportional LF (PLF) based on Equation (18) were performed to reconstruct phantoms respectively. To compare and analyze the accuracy of the reconstructed results, the following three quantitative metrics were adopted: image relative error (RE), size error (SE), and position error (PE). The RE assesses the error of the whole reconstructed image, expressed as

(27) $$\mathrm{RE}=\frac{∥\Delta \tilde{\sigma }-\Delta \sigma ∥}{∥\Delta \sigma ∥}$$

where $\Delta \tilde{\sigma }$ and Δσ are the reconstructed conductivity variation and the realistic conductivity variation, respectively. The smaller the value of RE, the better the image quality.

A $\frac{1}{4}$-amplitude set, $\Delta {\tilde{\sigma }}_{o}$, was calculated to extract observed anomaly objects, same as the process in Equation (10). The SE measures the extent to which reconstructed images represent the size of the object, expressed as

(28) $$\mathrm{SE}=\frac{|m_{o}^{\prime }-m_{o}|}{m_{o}}$$

where $m_{o}^{\prime }$ and $m_{o}$ are the numbers of anomaly object pixels of the reconstructed image and the ground truth, respectively. SE is desired to be low, for the aim of reliable interpretation of lung anomaly measurements.

PE measures the extent to which reconstructed images faithfully represent the position of the anomaly object, defined as

(29) $$\mathrm{PE}=∥r_{o}^{\prime }-r_{o}∥$$

where $r_{o}^{\prime }$ and $r_{o}$ represent the center of gravity of $\Delta {\tilde{\sigma }}_{o}$ and Δσ. PE should be low and ideally 0, which directly affects the accuracy of abnormal location diagnosis.

### 4.2. Image Reconstruction Using Simulated Noise-Free Data

The simulated imaging results (Case 1–6) are illustrated in Figure 5. Specifically, the first column exhibits the true conductivity distribution corresponding to different anomaly states, while the remaining columns exhibit the reconstruction results using different methods.

As shown in Figure 5, TK fails to detect local anomalies within the main objects. Using the proposed LF method, local anomalies can be reconstructed, and these anomalies in all cases are identifiable. However, without any prior conditions, FLF tends to over-reconstruct anomalies, resulting in ring artifacts around the objects that are hard to remove using the linearization algorithm. Moreover, there are fake anomalies in the form of artifacts, which might be caused by estimation errors in Layer 1.

As for the detection of small anomalies, narrower filters can be used to eliminate artifacts to improve the reconstruction accuracy. Additionally, if the lung contours are known, this can be used to directly build Layer 1, thereby promoting the reconstruction accuracy of Layer 2. It can be seen from the results of PLF that when some prior information is input to the algorithm, the new algorithm can almost completely restore the true distribution.

**Figure 6:**
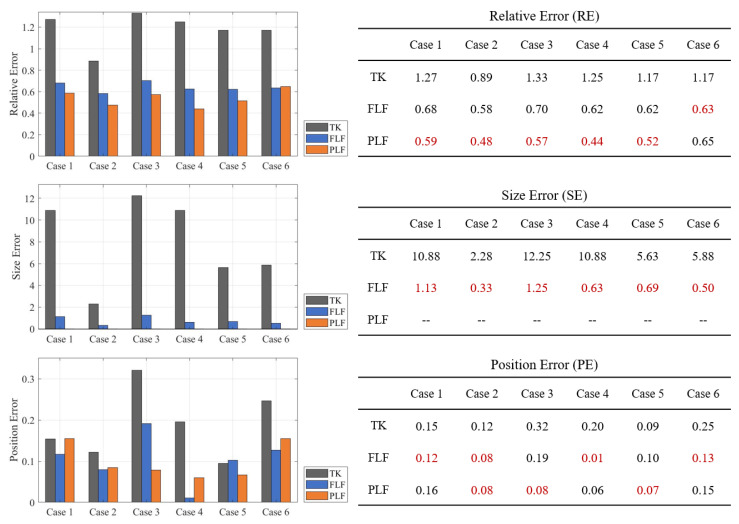
Relative error (RE), size error (SE), and position error (PE) across six different cases (Case 1 to Case 6) using three reconstruction methods: TK, FLF, and PLF. Those marked in red are with best performance.

Figure 6 illustrates the quantitative metrics of all reconstructed images in Figure 5, with the optimal method in each case marked in red. It can be concluded from RE that the overall accuracy can be significantly improved using the proposed methods of FLF and PLF. Using the PLF method based on prior information can achieve the best accuracy in most cases, while FLF is slightly worse than PLF in terms of metrics but exhibits significant improvements compared to TK, reducing the RE of TK by half.

The SE of TK results is very large in all six cases, which is consistent with the reconstruction result, indicating that TK cannot identify local anomalies. As discussed in the reconstruction result, adopting additional filters can enhance the accuracy of the reconstructed size. However, this also poses a risk of excessive size reductions, which may lead to a higher rather than a lower SE.

Compared with the overall RE and SE, the PE calculated through the center of gravity indicates that TK performs better in PE than in SE. This suggests that TK can detect local anomalies, but its one-step imaging capability is insufficient and cannot resolve the contours of local anomalies. After layered fusion, FLF can enhance both local anomaly detection and the location accuracy in most cases. However, PLF may not have the best position accuracy, despite its almost flawless image results.

### 4.3. Image Reconstruction Using Simulated Noisy Data

**Figure 7:**
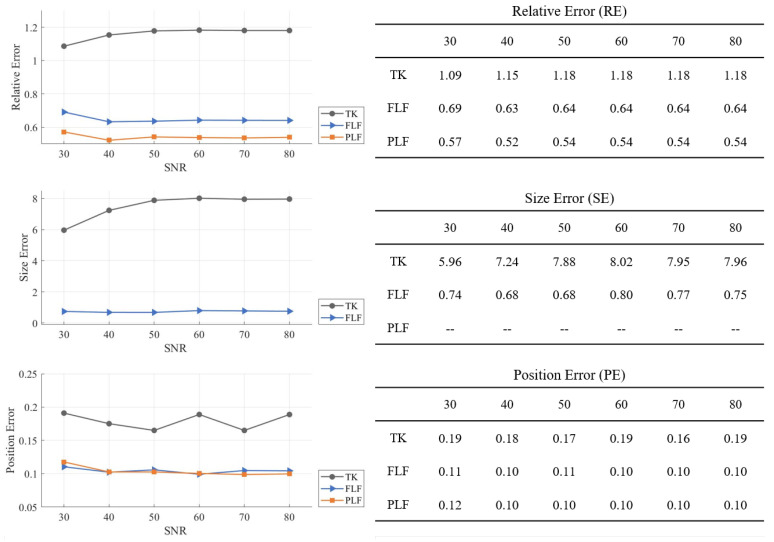
Average evaluation results under different SNR values and using different algorithms: TK, FLF, and PLF. Quantitative metrics are RE, SE and PE.

Figure 7 illustrates the average quantitative metrics of the reconstructed results for Cases 1–6, using data with a varying SNR. The results clearly indicate that both layered fusion methods (FLF and PLF) consistently outperform TK across all metrics. TK exhibits a high sensitivity to SNR variations, and the PE of TK does not positively correlate with changes in SNR. The proposed methods FLF and PLF are robust and stable under SNR changes, with PLF achieving the best RE.

### 4.4. Results of Experiments

A set of static tank experiments were carried out in previous work [17]. Data were collected using a TJU-EIT system using the adjacent measurement strategy, with an injection current of 5 mA and a frequency of 100 kHz. The system consists of a PC, an FPGA-based digital hardware system, and a tank equipped with a 16-electrode sensor system.

**Figure 8:**
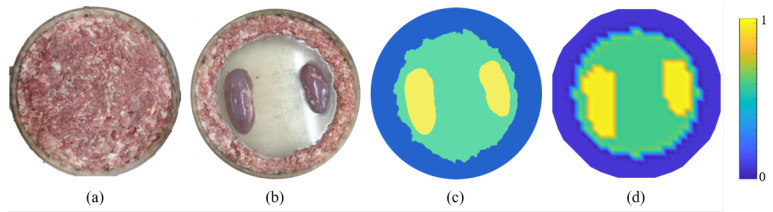
Experimental configuration and ground truth extraction for algorithm verification. (**a**) Reference scenario where tank was filled with chopped meat. (**b**) Observed domain that contains chopped meat, saline, and kidneys with varied conductivities. (**c**) Ground truth extracted from image processing. (**d**) Pixelated ground truth for reconstruction evaluation.

Figure 8 presents the configuration of the experiment, where (a) is the reference scenario where the tank was filled with chopped meat. Figure 8b illustrates the observed domain, where chopped meat was evenly placed on the outermost ring in the cylindrical tank to serve as the main background. Saline filled the inner area, serving as the primary imaging object. Kidneys were placed inside the saline to represent two anomaly areas. The conductivity values of the three media are related via $\sigma_{meat}<\sigma_{saline}<\sigma_{kedney}$. Figure 8c is the ground truth extracted by image processing on Figure 8b. Figure 8d is the ground truth directly exported on the simulation platform for reconstruction evaluation.

**Figure 9:**
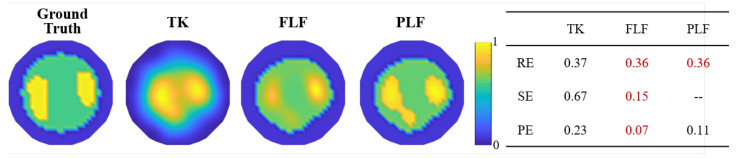
Visual reconstruction results and corresponding quantitative metrics in the experimental domain using TK, FLF, and PLF methods. Data marked in red are with best performance.

Figure 9 presents the reconstruction results obtained using the TK, FLF, and PLF algorithms. The corresponding quantitative metrics, RE, SE, and PE, of the experiment are also provided, offering a comprehensive evaluation of the performance of each algorithm.

As illustrated in Figure 9, the experiment yields similar results to the simulation in that TK struggles to accurately identify the object edge amidst the multi-conductivity distribution. This leads to significant discrepancies in the position of the anomaly. The application of FLF and PLF enhances differentiation of areas with varying conductivity values. It can be observed that the dimensions and locations of abnormal regions were determined with greater precision using FLF and PLF. All methods used for image reconstruction exhibit an abnormal protrusion in the upper direction on the left kidney. This anomaly could potentially be an error induced by the inherent non-uniformity of the meat ring. Judging by the quantitative metrics, FLF outperforms in terms of overall performance. While PLF uses a priori information to produce clear edges, it falls short in achieving an optimal positional accuracy, as shown in the PE.

In a comprehensive view, the use of prior information mainly impacts the visualization outcome. From the results, the performances of both FLF and PLF are largely analogous. Consequently, if there is a lack of sufficient prior conditions, the filter-based layered fusion method, FLF, can be directly implemented. It is worth noting that all computations incorporated in this method are fundamentally rooted in the principles of EIT and do not require any additional parameter adjustment procedures. Therefore, it stands as a robust, uncomplicated, and relatively accurate reconstruction method.

## 5. Discussion

A multi-conductivity EIT layered fusion reconstruction frame, LF, is proposed. LP can be divided into FLF and PLF according to whether the local proportion is prior or not. LF is derived based on the internal relation of EIT and fuzzy features in EIT images. This frame is easy to calculate, does not introduce an uncertainty coefficient, and does not require iterations. The results show that the current method significantly improves the accuracy of EIT imaging under different conductivity conditions. The simplicity and accuracy of the method are critical for the reconstruction of local anomalies in multi-conductivity distributions in clinical diagnosis and industrial monitoring. The results show that the current method significantly improves the accuracy of EIT imaging under multi-conductivity conditions. The simplicity and accuracy of the method are critical for the reconstruction of local anomalies in multi-conductivity distributions in clinical diagnosis and industrial monitoring.

It is essential to recognize that LF is primarily limited to cases where a three-phase distribution is present. It is assumed that there are local anomalies inside the objects, and the conductivity characteristics of the abnormal region are uniform. For scenarios that extend beyond this predefined range, alternative considerations are necessary. In cases where the subject is free from disease or where only two phases of conductivity are present, traditional EIT reconstruction methods or their advanced patterns are typically sufficient [30].

The proposed method is designed to enhance imaging in more complex situations where traditional approaches may fall short. While our current method is optimized for three layers, it can be extended to accommodate more layers, thereby addressing scenarios with more than three conductivities. However, the method in its current form does not inherently determine the number of layers or phases present. Future work could involve the development of an algorithmic coefficient that can dynamically assess and adapt to the number of conductive phases, thereby enhancing the versatility of the method. This would enable our method to be applied in a broader range of scenarios, including those with an indeterminate number of conductivities. Additionally, the robustness of the method should be tested across a wider variety of conditions to ensure its reliability and accuracy.

Deep learning has emerged as a promising approach in medical imaging, with numerous models developed for lung detection. However, the majority of deep learning research in EIT has been concentrated on the study of non-local lesions and the identification of dispersed multi-conductivity objects [15,34,35,36]. While these models have demonstrated the potential to achieve over 90% accuracy in EIT reconstruction for the aforementioned scenarios, the detection of local anomalies remains a significant challenge. A critical challenge in leveraging deep learning for local lesion detection is the reliance on the quality and availability of clinical datasets, which are notoriously difficult to acquire. Furthermore, the training process for deep learning models is time-consuming and resource-intensive. Although no definitive solution exists for local anomaly reconstruction, the potential of deep learning to improve EIT is significant and merits further research and development.

At present, EIT serves as a complementary imaging modality, particularly useful in cases where a disease has already been identified or is suspected. The value of EIT lies in its ability to elucidate internal relationships within the context of some prior knowledge. Therefore, the pursuit of simple yet effective methods that balance ease of use with imaging accuracy represents a meaningful direction for the advancement of EIT. The development of such methods could significantly enhance the utility of EIT in clinical settings.

In conclusion, while layered fusion reconstruction represents an advancement for EIT in multi-conductivity scenarios, it is not without its limitations. The assumptions made regarding the number and uniformity of conductive phases may restrict its applicability in certain contexts. Nevertheless, it is optimistic that with further refinement and the incorporation of adaptive algorithms, the proposed approach can be expanded to meet a wider array of clinical and industrial needs.

## 6. Conclusions

A novel layered fusion reconstruction framework for multi-conductivity EIT is proposed in this paper. The proposed approach effectively addresses the challenge of detecting local anomalies. By initiating with pre-imaging and extracting primary objects from the blurred image, Layer 1 is established for further analysis. The subsequent estimation of the remaining voltage differences and the construction of the corresponding conductivity in Layer 2 further enhance the detection capabilities of local anomalies. The integration of multiple layers and the exploration of inherent electrical impedance characteristics make it possible to reconstruct multiple conductivity distributions. Validated through rigorous simulations and experiments, the proposed method has demonstrated significant improvements in multi-layer separation, precise anomaly localization, and overall robustness when compared to the traditional regularization method. The proposed method paves the way for future works aiming to further improve the detection and analysis of local anomalies in biological tissues in a fast and simple way. In future work, further studies will be conducted to simulate more complex scenarios, conduct clinical experiments, quantify improvements over traditional methods, and explore potential clinical applications.

## Data Availability

The raw data supporting the conclusions of this article will be made available by the authors on reasonable request.

## References

[1] Cui Z., Zhang Q., Gao K., Xia Z., Wang H. (2021). Electrical impedance sensors for multi-phase flow measurement: A review. IEEE Sens. J..

[2] Ke X.Y., Hou W., Huang Q., Hou X., Bao X.Y., Kong W.X., Li C.X., Qiu Y.Q., Hu S.Y., Dong L.H. (2022). Advances in electrical impedance tomography-based brain imaging. Mil. Med. Res..

[3] Gomes J.C., Barbosa V.A., Ribeiro D.E., de Souza R.E., dos Santos W.P. (2020). Electrical impedance tomography image reconstruction based on backprojection and extreme learning machines. Res. Biomed. Eng..

[4] Liu D., Khambampati A.K., Kim S., Kim K.Y. (2015). Multi-phase flow monitoring with electrical impedance tomography using level set based method. Nucl. Eng. Des..

[5] Zhao Y., Yue S., Zhang Y., Wang H. (2023). Flow velocity computation using a single ERT sensor. Flow Meas. Instrum..

[6] He H., Jiang J., Xu M., Yuan S., Long Y., Chi Y., Frerichs I., Zhao Z. (2022). Saline bolus-based electrical impedance tomography method for rapid bedside assessment of regional lung perfusion during ECMO therapy. Crit. Care.

[7] Shi Y., Yang Z., Xie F., Ren S., Xu S. (2021). The research progress of electrical impedance tomography for lung monitoring. Front. Bioeng. Biotechnol..

[8] Park H., Park K., Mo S., Kim J. (2021). Deep neural network based electrical impedance tomographic sensing methodology for large-area robotic tactile sensing. IEEE Trans. Robot..

[9] Nawaz M., Chan R.W., Malik A., Khan T., Cao P. (2022). Hand Gestures Classification Using Electrical Impedance Tomography Images. IEEE Sens. J..

[10] Zheng Z., Wu Z., Zhao R., Ni Y., Jing X., Gao S. (2022). A review of EMG-, FMG-, and EIT-based biosensors and relevant human–machine interactivities and biomedical applications. Biosensors.

[11] Xu M., He H., Long Y. (2021). Lung perfusion assessment by bedside electrical impedance tomography in critically ill patients. Front. Physiol..

[12] Barbas C.S., Amato M.B. (2022). Electrical Impedance Tomography to Titrate PEEP at Bedside in ARDS. Respir. Care.

[13] Gabriel S., Lau R., Gabriel C. (1996). The dielectric properties of biological tissues: II. Measurements in the frequency range 10 Hz to 20 GHz. Phys. Med. Biol..

[14] Adler A., Boyle A. (2017). Electrical impedance tomography: Tissue properties to image measures. IEEE Trans. Biomed. Eng..

[15] Zhang K., Guo R., Li M., Yang F., Xu S., Abubakar A. (2020). Supervised descent learning for thoracic electrical impedance tomography. IEEE Trans. Biomed. Eng..

[16] Becher T., Bußmeyer M., Lautenschläger I., Schädler D., Weiler N., Frerichs I. (2018). Characteristic pattern of pleural effusion in electrical impedance tomography images of critically ill patients. Br. J. Anaesth..

[17] Sun B., Yue S., Hao Z., Cui Z., Wang H. (2019). An improved Tikhonov regularization method for lung cancer monitoring using electrical impedance tomography. IEEE Sens. J..

[18] Wang Q., Wang H. (2011). Image reconstruction based on 11 regularization for electrical impedance tomography (EIT). Proceedings of the 2011 IEEE International Instrumentation and Measurement Technology Conference.

[19] Wang Q., Lian Z., Wang J., Chen Q., Sun Y., Li X., Duan X., Cui Z., Wang H. (2016). Accelerated reconstruction of electrical impedance tomography images via patch based sparse representation. Rev. Sci. Instrum..

[20] Fan Y., Ying L. (2020). Solving electrical impedance tomography with deep learning. J. Comput. Phys..

[21] Aller M., Mera D., Cotos J.M., Villaroya S. (2023). Study and comparison of different Machine Learning-based approaches to solve the inverse problem in Electrical Impedance Tomographies. Neural Comput. Appl..

[22] Wang Z., Zhang X., Fu R., Wang D., Chen X., Wang H. (2023). Electrical Impedance Tomography Image Reconstruction with Attention-based Deep Convolutional Neural Network. IEEE Trans. Instrum. Meas..

[23] Hamilton S.J., Hauptmann A. (2018). Deep D-bar: Real-time electrical impedance tomography imaging with deep neural networks. IEEE Trans. Med. Imaging.

[24] Capps M., Mueller J.L. (2020). Reconstruction of organ boundaries with deep learning in the D-bar method for electrical impedance tomography. IEEE Trans. Biomed. Eng..

[25] Rymarczyk T., Kłosowski G., Kozłowski E., Tchórzewski P. (2019). Comparison of selected machine learning algorithms for industrial electrical tomography. Sensors.

[26] Adler A., Arnold J.H., Bayford R., Borsic A., Brown B., Dixon P., Faes T.J., Frerichs I., Gagnon H., Gärber Y. (2009). GREIT: A unified approach to 2D linear EIT reconstruction of lung images. Physiol. Meas..

[27] Tomicic V., Cornejo R. (2019). Lung monitoring with electrical impedance tomography: Technical considerations and clinical applications. J. Thorac. Dis..

[28] Prins S.A., Weller D., Labout J.A., den Uil C.A. (2023). Electrical Impedance Tomography as a Bedside Diagnostic Tool for Pulmonary Embolism. Crit. Care Explor..

[29] Shin K., Ahmad S., Santos T.B.R., Barbosa da Rosa Junior N., Mueller J.L. (2023). Comparison of Two Linearization-Based Methods for 3-D EIT Reconstructions on a Simulated Chest. J. Math. Imaging Vis..

[30] Wang Z., Liu X. (2023). A regularization structure based on novel iterative penalty term for electrical impedance tomography. Measurement.

[31] Wang Z., Sun Y., Li J. (2024). Posterior Approximate Clustering-Based Sensitivity Matrix Decomposition for Electrical Impedance Tomography. Sensors.

[32] Kang S.I., Khambampati A.K., Jeon M.H., Kim B.S., Kim K.Y. (2016). A sub-domain based regularization method with prior information for human thorax imaging using electrical impedance tomography. Meas. Sci. Technol..

[33] Bai X., Liu D., Wei J., Bai X., Sun S., Tian W. (2021). Simultaneous Imaging of Bio-and Non-Conductive Targets by Combining Frequency and Time Difference Imaging Methods in Electrical Impedance Tomography. Biosensors.

[34] Cheng Y., Fan W. (2022). R-UNet deep learning-based damage detection of CFRP with electrical impedance tomography. IEEE Trans. Instrum. Meas..

[35] Zhang T., Tian X., Liu X., Ye J., Fu F., Shi X., Liu R., Xu C. (2022). Advances of deep learning in electrical impedance tomography image reconstruction. Front. Bioeng. Biotechnol..

[36] Liu D., Wang J., Shan Q., Smyl D., Deng J., Du J. (2023). DeepEIT: Deep image prior enabled electrical impedance tomography. IEEE Trans. Pattern Anal. Mach. Intell..

